# Electrode Layout Optimization and Numerical Simulation of Cast Conductive Asphalt Concrete Steel Bridge Deck Pavement

**DOI:** 10.3390/ma15197033

**Published:** 2022-10-10

**Authors:** Zhenxia Li, Tengteng Guo, Yuanzhao Chen, Wenping Yang, Shengquan Ding, Menghui Hao, Xu Zhao, Jinyuan Liu

**Affiliations:** 1 School of Civil Engineering and Communication, North China University of Water Resources and Electric Power, Zhengzhou 450045, China; 2 Henan Province Engineering Technology Research Center of Environment Friendly and High-Performance Pavement Materials, Zhengzhou 450045, China; 3 Zhengzhou City Key Laboratory of Environmentally Friendly High Performance Road and Bridge Materials, Zhengzhou 450045, China; 4 Henan Communications Planning & Design Institute Co., Ltd., Zhengzhou 450046, China

**Keywords:** road engineering, electrode layout optimization, cast conductive asphalt concrete, finite element analysis, orthotropic steel deck, melting ice and snow

## Abstract

In order to obtain the optimal electrode layout and ice melting effect of cast conductive asphalt concrete steel bridge deck pavement, firstly, pouring conductive asphalt concrete was prepared; secondly, different electrode materials and layout methods were selected to test the heating rate of the specimen from start to 120 min, and the electrode materials and layout methods were optimized. Then, the finite element analysis software ANSYS was used to build the model for heating and ice melting simulation, and the indoor test was used to further verify the ice melting effect of the cast conductive asphalt coagulation with or without the insulation layer. Finally, the thermal-structural coupling analysis of cast conductive asphalt concrete steel bridge deck pavement was carried out using ANSYS finite element software. The results showed that the stainless steel electrode material had the best heating effect, and the electrode thickness in the range of 0.1~3mm had no effect on the heating effect. The intermediate heating rate of the upper surface of the stainless steel sheet electrode cast conductive asphalt concrete in the left and right external electrodes was 8 °C/h, while the intermediate heating rate of the upper surface of the stainless steel mesh electrode cast conductive asphalt concrete was 12.9 °C/h. The layout of the left and right buried stainless steel metal mesh was able to effectively improve the snow melting efficiency; ANSYS finite element ice melting simulation was used to obtain the variation law of ice melting efficiency and a temperature field of cast conductive asphalt concrete. The indoor ice melting test showed that when melting the same thickness ice layer at 50 V voltage, it took 240 min with an insulation layer and 720 min without an insulation layer, which was three times that of the ice with an insulation layer, which further verifies the superiority of its ice melting effect. The most unfavorable load position of pavement under load and temperature field was determined. The maximum tensile stress and compressive stress of the pavement surface were transverse, and the maximum shear stress of the pavement bottom was transverse.

## 1. Introduction

The proportion of China’s land area in the cold region is large, and there is snow and ice in winter [1]. Grouting in the asphalt concrete steel bridge deck also has the problem of icing. Snow and ice on the bridge deck not only hinder traffic, but may also pose a threat to the safety of life and property. In the face of severe problems such as snow and ice on the road area in winter, the traditional de-icing method of the road consumes a lot of energy and has low snow-removal efficiency [2,3,4]. It will also corrode the road infrastructure and pollute the environment and the building itself, resulting in high repair costs [5,6,7,8]. Although scholars in various countries have carried out a lot of research on cast conductive asphalt concrete, there are few articles on electrode materials and layout methods in conductive concrete, and there are few mechanical studies on the thermal-structural coupling of steel bridge deck pavement.

As a new type of snow-removal technology, pouring conductive asphalt concrete has the advantages of high de-icing efficiency, good compliance with the bridge deck, no water seepage and low energy consumption [9,10,11,12]. The Nebraska Department of Roads conductive concrete bridge de-icing project analyzed the cost of various de-icing methods. It was found that the cost of conductive concrete was the lowest among different heating and snow melting methods, followed by the electric wire method. At the same time, many domestic researchers also believe that conductive concrete is the most economical and effective de-icing method for bridge deck [13,14]. Its total energy consumption per unit area is expected to be applied to special sections such as bridge decks, highways and even airport pavements with harsh snow-removal requirements, with considerable economic benefits. Wang C.H. [15,16,17] prepared cast asphalt concrete which met the construction workability, high temperature stability and electrical conductivity requirements at the same time. The road performance and snow melting effect of different types of cast conductive asphalt concrete were systematically studied. The use of different electrode materials and layout methods of conductive concrete and the resistance generated by them will interfere with the heating rate of concrete slabs. The concept of embedded mesh electrodes was gradually proposed as the test progressed. The layout of the upper and lower mesh electrodes was closely related to the concrete, which reduced the contact resistance and the heating rate of the concrete slab was higher [18,19]. Tang Z.Q., Q J.S. et al. [20] found that the electrode materials and electrode layout form were different, which affected the heating rate and temperature distribution of the concrete slab. From the contact resistance, it is explained that the contact resistance between the embedded stainless steel plate electrode and the concrete is small, and the contact resistance between the external stainless steel plate electrode and the concrete is not close enough, which leads to the contact resistance being large, so that the resistivity of the conductive concrete with the stainless steel metal mesh electrode is low, the heating rate is fast, and the cost can be reduced under the condition of ensuring the strength of the concrete. The conductive asphalt concrete specimen with steel sheet electrode has the lowest resistance. The smaller the spacing is, the smaller the resistance is, and the larger the contact area, the more conductive paths are formed and the better the conductive effect [21,22,23,24]. The electrode material type, layout form, material characteristics and measurement methods will affect the resistivity measurement results of conductive concrete [25,26,27].

In summary, at this stage, the research on cast conductive asphalt concrete steel bridge deck pavement is more comprehensive, and has achieved good results in practical application. The resistivity of conductive asphalt concrete and the ways to reduce the contact resistance have also made significant progress. However, the electrode materials used for conductive asphalt concrete are relatively simple, and the electrode layout is not comprehensive. In order to expand the snow-melting and ice-melting effect of cast-in-place conductive asphalt concrete steel bridge deck pavement, this paper first prepared cast-in-place conductive asphalt concrete, and then selected different electrode materials and layout methods. The best electrode materials and layout methods were selected by conducting an electric heating test of cast-in-place conductive asphalt concrete. On this basis, the finite element simulation of ice melting was carried out, and the ice melting research was carried out in the laboratory. Finally, the internal stress and strain of cast-in-place conductive asphalt concrete steel bridge deck under ice melting conditions were analyzed.

## 2. Materials and Methods

### 2.1. Asphalt

The asphalt used in this paper is SBS modified asphalt from Zhengzhou Zhengfa Municipal Construction Co., Ltd. (Beijing, China), which belongs to I-D modified asphalt. Its basic technical indicators and requirements are shown in Table 1.

### 2.2. Aggregates and Mineral Powder

The aggregate used for this paper is basalt rolling crushed stone, and the mineral powder is limestone powder. The amount is about 20–30%, or even higher, while the amount of asphalt is approximately 8–12%. The performance indexes of aggregate and mineral powder are shown in Table 2, Table 3 and Table 4.

### 2.3. Conductive Materials

It has been found that carbon fiber length of 9 mm has a good dispersion effect; therefore this study selected flake graphite, and carbon fiber length of 9 mm. For graphite and carbon fiber technical indicators, see Table 5 and Table 6.

### 2.4. Electrode Material

The electrode materials are mainly divided into metal materials and non-metal materials. The metal electrode is a metal material with a bare surface and strong conductive ability. Metal material has high strength, and the surface-treated metal has stronger corrosion resistance. Compared with non-metallic materials, a metal electrode is easier to prepare and lay out. Therefore, metal electrodes are selected for layout comparison.

Commonly used metal electrode materials are copper, iron, stainless steel, zinc and aluminum. The material properties are shown in Table 7.

### 2.5. Preparation of Pouring Conductive Asphalt Concrete

The comparison of electrodes requires the preparation of cast conductive asphalt concrete. The gradation selection of cast conductive asphalt mixture used in this paper is shown in Table 8.

**Figure 1:**
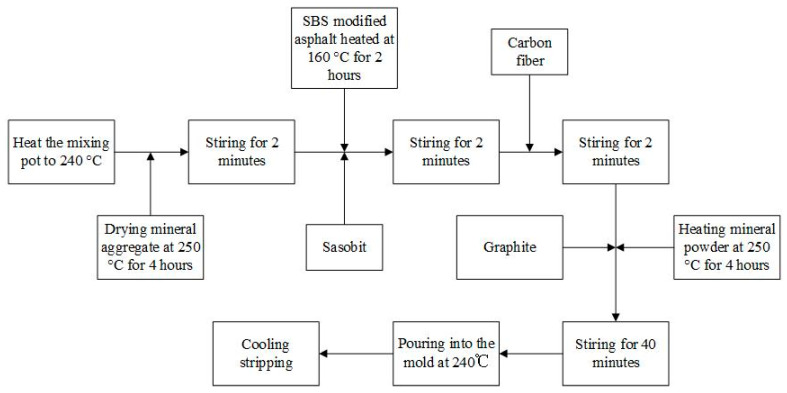
Preparation process of CGA-10.

The dosage of pouring conductive asphalt concrete is: SBS modified asphalt dosage 13.2%, Sasobit modifier 3% (asphalt mass fraction), graphite 40% (equal volume instead of mineral powder), carbon fiber dosage 0.4% (according to the mass percentage of mineral). The preparation process is shown in Figure 1.

### 2.6. Electrode Layout Form

**Figure 2:**
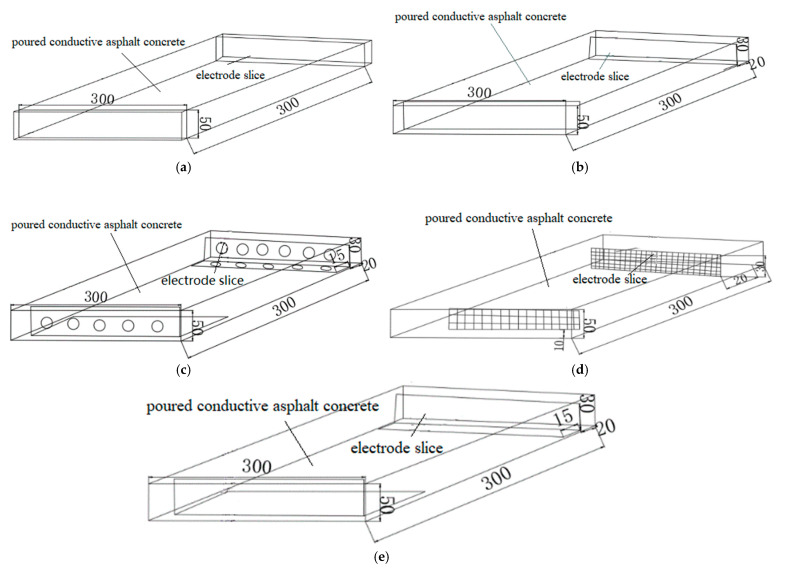
Design drawing of electrode layout scheme. (**a**) Layout of left and right electrodes; (**b**) Direct electrode embedded in left and right; (**c**) L electrode embedded with holes; (**d**) Left and right embedded mesh electrodes; (**e**) L electrode buried left and right.

The electrode layout has a significant impact on the thermoelectric effect of conductive asphalt concrete. The electrodes are arranged in two ways: external and internal. The external are left and right external. The sheet electrode with an electrode size of 300 mm × 50 mm is selected. The internal is left and right internal. The L-shaped hole and no-hole and mesh electrodes are selected. The electrode arrangement scheme is shown in Figure 2.

#### 2.6.1. Left and Right External Electrode Layout

**Figure 3:**
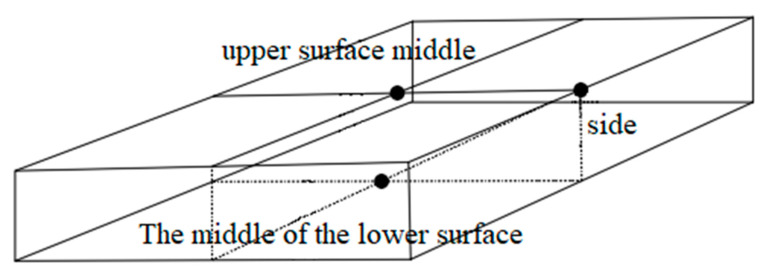
Layout of temperature sensor.

**Figure 4:**
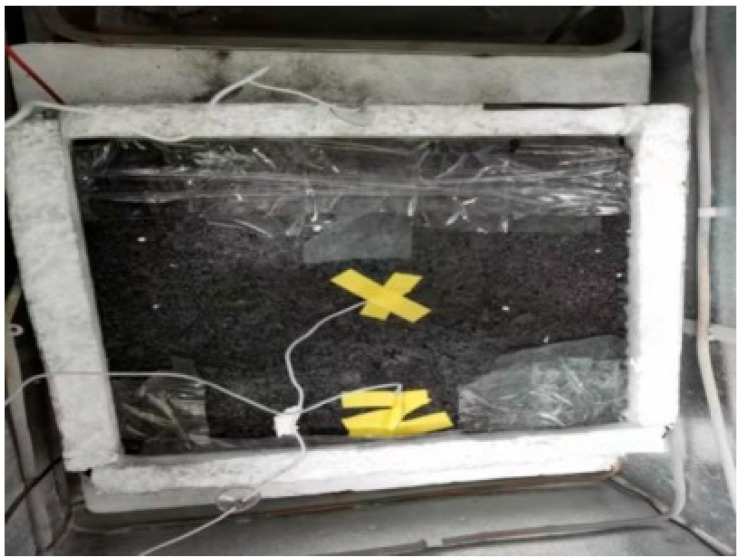
Placement of test piece.

**Figure 5:**
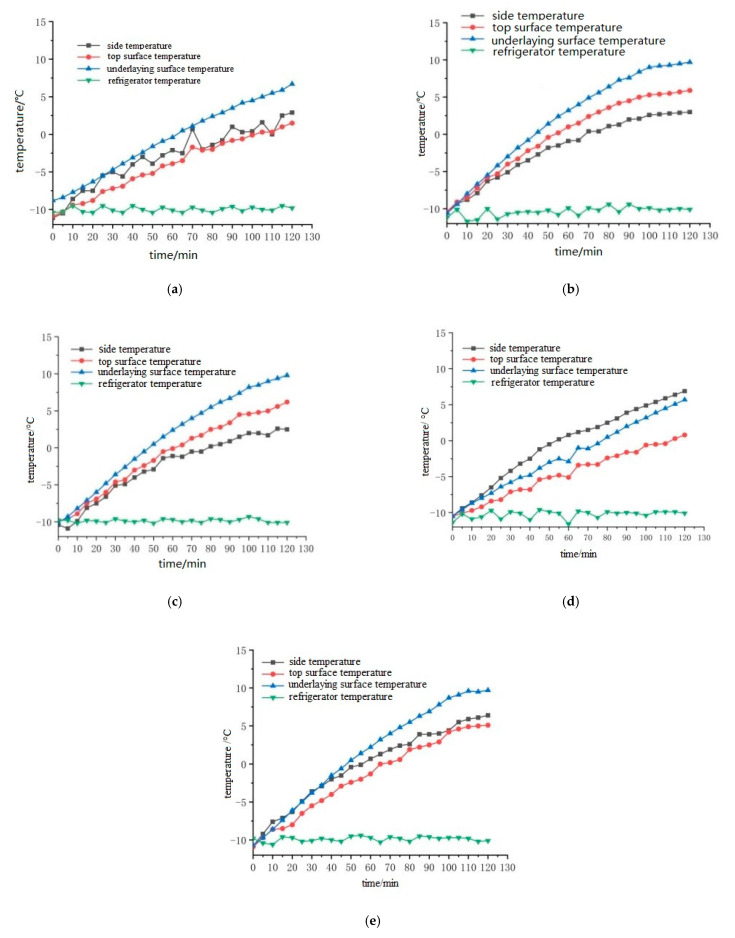
Heating diagram of different electrodes. (**a**) Heating of 0.1 mm thick externally bonded aluminum sheet; (**b**) Heating of 0.1 mm thick externally bonded zinc sheet; (**c**) Heating of 0.1 mm thick externally bonded copper sheet; (**d**) Heating of 0.1 mm thick externally bonded iron sheet; (**e**) Heating of 0.1 mm thick externally bonded stainless steel sheet.

**Figure 6:**
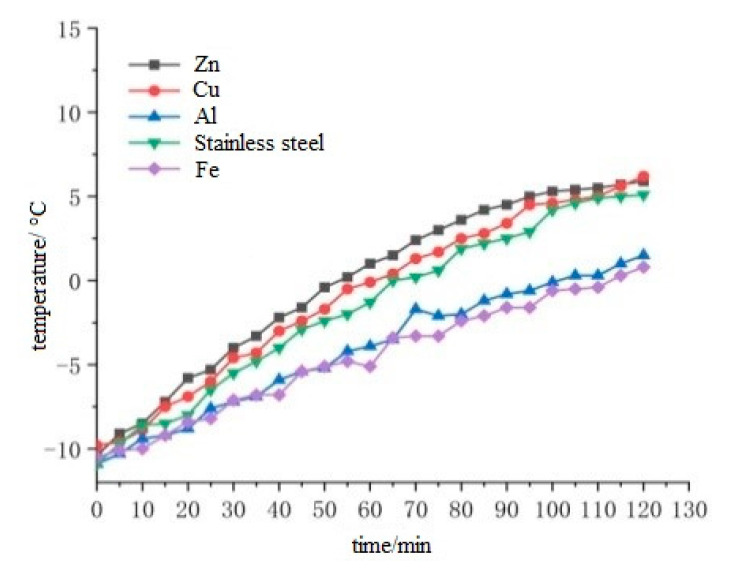
Diagram of the influence of different electrode sheets on the heating rate.

In order to reduce the influence of contact resistance, graphite is spread on the side of the specimen. The electrode plates are copper, aluminum, iron, stainless steel and zinc, and the sizes are 300 mm × 50 mm × 0.1 mm. The electrode plates are welded to the wire. After embedding the temperature sensor, the pouring conductive asphalt concrete with electrodes is wrapped with foam insulation material, and then the electric heating test is carried out. The temperature sensor is embedded as shown in Figure 3, and placed in a low temperature box. The initial temperature is set to −10 °C. After 12 h of placement, the 50 V voltage is connected, and the electricity is electrified for 120 min. The temperature of the side, the middle of the upper surface and the middle of the lower surface of the specimen is recorded every 5 min. The placement of the specimen is shown in Figure 4, and the temperature rise is shown in Figure 5 and Figure 6.

It can be seen from Figure 5 that under the condition of 50 V power supplies, the middle temperature of the upper surface and the middle temperature of the lower surface of the cast conductive asphalt concrete with the external electrode are basically linear. From Figure 6, it can be seen that the temperature of the zinc sheet electrode at each time point is above the temperature of the other electrode time points. It can be seen that the heating rate of the specimen is zinc sheet electrode, copper sheet electrode, stainless steel sheet electrode, aluminum sheet electrode and iron sheet electrode. It can be seen from Table 8 that the cost of electrode plates from high to low is zinc, copper, aluminum, stainless steel and iron. Taking into account the large rigidity of the iron and aluminum sheets, large contact resistance will be produced under external conditions, which affects the heating effect. Although zinc sheet has a good heating effect and durability, the cost is too high. The heating effect of the copper electrode is second only to zinc sheet. After two hours of heating, it also has a rapid upward trend, but the price is higher. The stainless steel sheet with a similar heating effect is cheaper. Considering the conductivity, heating performance, durability and cost, the stainless steel sheet electrode was selected as the external electrode, and stainless steel was selected as the electrode material.

#### 2.6.2. Layout of Left and Right Embedded Electrodes

For the left and right buried electrodes, the greater the coverage of the electrode in conductive asphalt concrete, the more conductive pathways, and the better the heating effect. The electrodes are divided into the following five electrode forms when the left and right electrodes are buried: (1) the electrode height is designed to be 25 mm, and the electrode width is designed to be 250 mm, in the form of straight and mesh electrodes. (2) The electrode sizes of L-type straight plate with and without holes are 25 mm in upper L height, 25 mm in lower L length and 250 mm in width. (3) The length and width of the full thickness electrode with holes is 300 mm length × 50 mm width. The number of holes is arranged according to the average electrode length, and the aperture is 13 mm.

**Figure 7:**
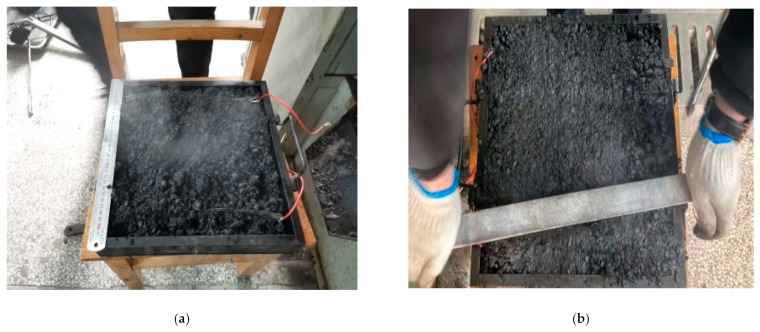
Left and right mesh and straight electrode layout. (**a**) mixture paving; (**b**) Surface smoothing.

The layout process of the stainless steel straight electrode and the mesh electrode is to lay conductive asphalt concrete 15 mm at the bottom of the test die, then lay the electrode 20 mm away from the edge of the test die, and cast the electrode to 50 mm. The L-type electrode with holes and the L-type electrode without holes is first poured 15 mm conductive asphalt concrete at the bottom of the test die. The electrode is then laid on the pouring conductive asphalt concrete, and the conductive asphalt concrete is poured to 50 mm thicknesses. The full-thickness electrode plate with holes is first embedded in the test mold, and the conductive asphalt concrete is slowly poured to full thickness. The specimen molding is shown in Figure 7.

**Figure 8:**
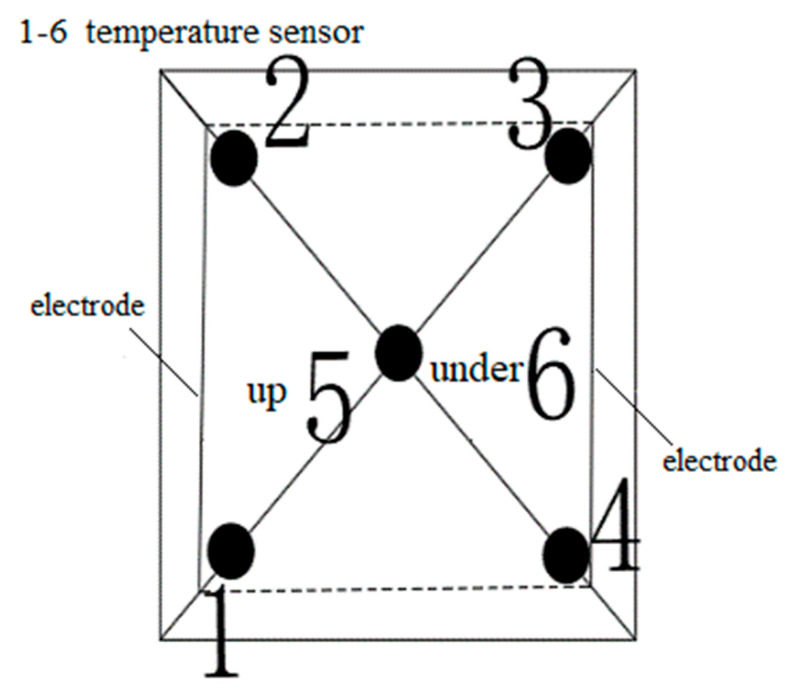
Layout of buried electrode temperature sensor.

**Figure 9:**
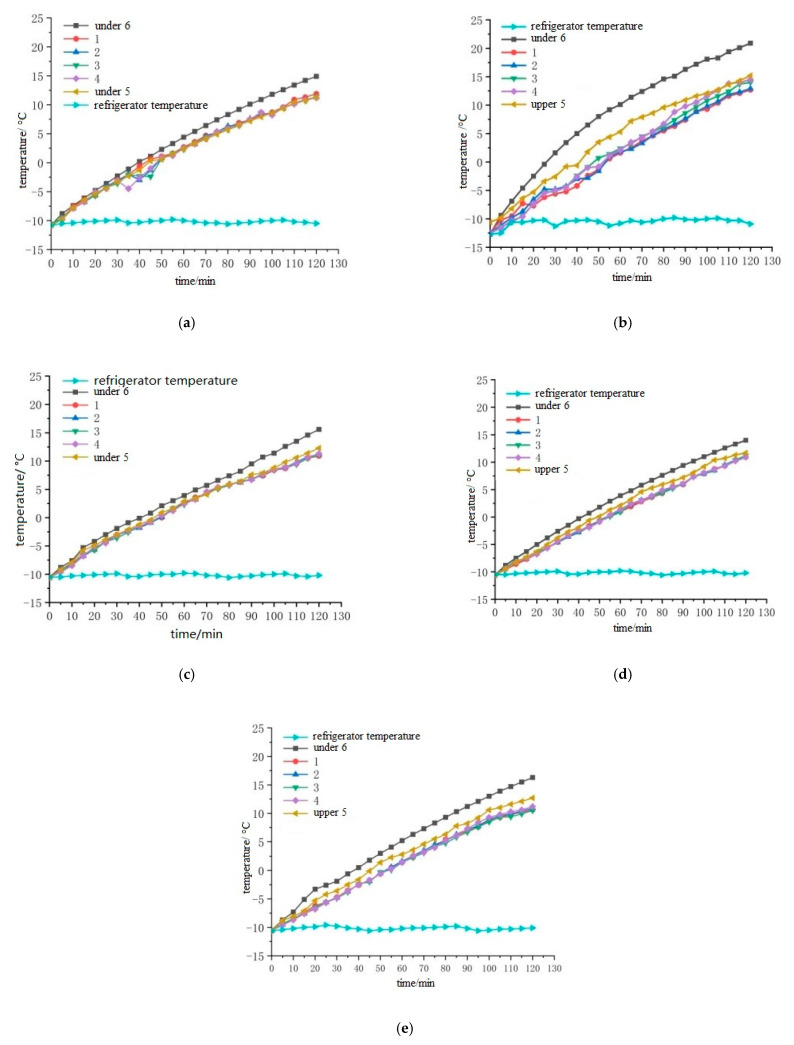
The heating curve of the left and right buried electrodes. (**a**) Temperature rise diagram of left and right straight electrode; (**b**) Temperature rise diagram of left and right mesh electrode; (**c**) Temperature rise diagram of left and right L-shaped electrode with hole; (**d**) Temperature-rise diagram of left and right L-shaped electrode; (**e**) Temperature rise diagram of full-thickness electrode with holes.

**Figure 10:**
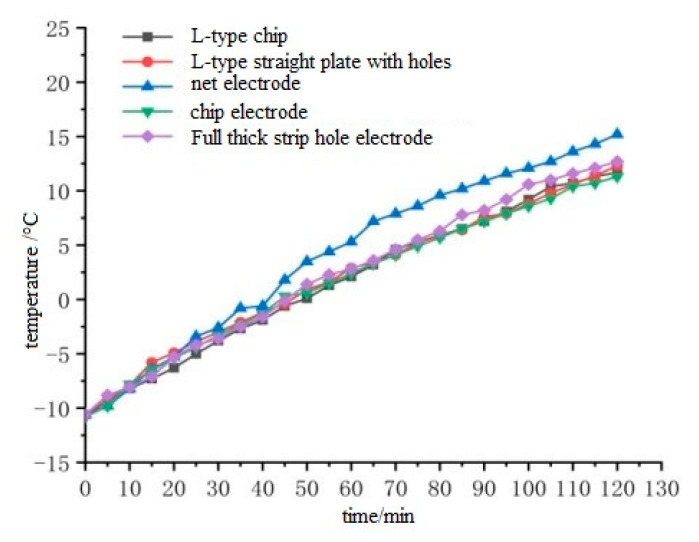
Comparison of the temperature rise of the upper surface of the buried electrodes on the left and right sides.

The pouring conductive asphalt concrete specimen is wrapped with polyethylene foam, and the temperature sensor is placed in the low temperature box. The layout of the temperature sensor is shown in Figure 8. The results of temperature rise are shown in Figure 9 and Figure 10.

From Figure 9, it can be seen that the temperature rise of each point on the surface of cast conductive asphalt concrete is similar, and the middle temperature is the highest, which is due to the good contact between the embedded electrode and the conductive asphalt concrete, and the contact resistance is smaller. It can be seen from Figure 10 that in the comparison of the temperature rise of the left and right buried electrodes, the temperature rise of the left and right mesh buried electrodes is the fastest, followed by the left and right full-thickness buried electrodes with holes, the left and right L-shaped buried electrodes with holes, the left and right L-shaped straight buried electrodes, and the left and right straight buried electrodes. It can also be seen that the left and right mesh buried electrodes have the best heating effect. By comparing the left and right L-shaped straight buried electrodes with holes and the left and right L-shaped buried electrodes with holes, it can be seen that the hole electrode can make the pouring conductive asphalt concrete have a better heating effect, which is because the asphalt concrete is connected through the holes, so that the electrode and asphalt concrete are closer. However, the design steps of the left and right L-shaped buried electrodes with holes are complex and not suitable for construction. In order to obtain more conductive pathways, the heating effect of the full-thickness embedded electrode with holes is second only to that of the mesh embedded electrode. This is because the full-thickness electrode covers a large surface and forms many conductive pathways. The asphalt mixture is connected through holes, and the contact resistance is reduced by squeezing the electrode. However, the electrode is slightly exposed to air, affecting the service life. At the same time, there are security risks, and the design of the electrode is also complex. In summary, the left and right mesh electrodes are selected for the layout of the left and right buried electrodes. Due to the low heating rate of the external electrode and the need to spread the excessive layer, the cost is increased. Therefore, considering the service life of roads and bridges and the engineering cost, the stainless steel metal mesh electrode embedded in the left and right is selected as the optimal electrode layout.

### 2.7. Finite Element Analysis

The finite element calculation software ANSYS is used to verify the temperature rise test of the pouring conductive asphalt concrete, and the effect of the electrode layout optimization is further clarified. The finite element simulation of the melting ice and snow of the pouring conductive asphalt concrete is then carried out. In line with the indoor melting ice and snow test, the reliability of the finite element simulation of the melting ice effect of the pouring conductive asphalt concrete is verified, which reflects the effect of the electrode layout optimization scheme on the actual pavement melting ice.

#### 2.7.1. Parameter Determination of Finite Element Analysis

The latent heat is often taken into account in the phase change analysis of ice. The enthalpy of ice at various temperatures is shown in Table 9, and the ANSYS finite element analysis parameters are selected as shown in Table 10.

#### 2.7.2. Finite Element Simulation of Ice-Snow Melting of Pouring Conductive Asphalt Concrete Slab

Under the condition of temperature change and electric conduction, the resistivity of electrodes and castable conductive asphalt concrete will change to some extent. Contact resistance will also be generated, due to the contact tightness between electrodes and wires and between electrodes and castable conductive asphalt concrete, which will bring a huge amount of calculation to the model processing. In order to simplify the model and reduce the amount of calculation, the following assumptions are made according to the actual situation: (1) various materials used in the model are homogeneous and continuous materials. (2) Without considering the contact resistance between the electrode and the pouring conductive asphalt concrete, the contact between the two solids is close. (3) Without considering the contact thermal resistance between the electrode and the pouring conductive asphalt concrete, the contact between the two solids is close. (4) Each physical parameter does not change with the change of temperature, which is a fixed value and does not consider the influence of low temperature box radiation. (5) The ice layer is homogeneous and isotropic material, and the melting process of the ice layer is reflected by temperature, without considering the evaporation in the melting process of the ice layer. (6) The steel plate material is isotropic and the bonding between the structural layers is good.

**Figure 11:**
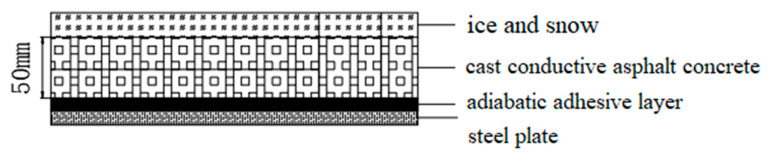
Casting conductive asphalt concrete ice melting model.

The finite element thermal analysis environment temperature is set to −10 °C, and the pouring conductive asphalt concrete melting ice and snow model is shown in Figure 11. The SOLID226 thermoelectric analysis unit is selected for the cast conductive asphalt concrete layer and electrode, and the SOLID70 thermal analysis unit is selected for the ice layer and the stainless steel bottom plate. The convective heat transfer coefficient between the surface of the cast conductive asphalt concrete and the air is 6.02 W/m^2^·K, and the convective heat transfer coefficient between the stainless steel and the air is 10 W/m^2^·K when natural convection occurs. The latent heat of the melting process of the ice block is reflected in the enthalpy change value, without considering radiation.

**Figure 12:**
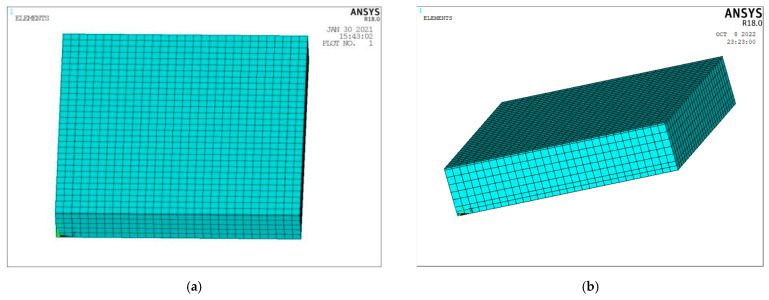
Mesh division of the melting ice model. (**a**) Grid division with heat insulation model; (**b**) Meshing without heat insulation model.

In the finite element modeling with an insulation layer, because the bottom plate means that the insulation is good, the insulation adhesive layer and steel plate are omitted, and the conductive asphalt concrete layer and ice layer only are established. The convective heat transfer coefficient of ice and air is 34 W/m^2^·K. When there is no insulation analysis, the insulation layer is removed, and the convective heat transfer is applied to the outer surface of the model, without considering the thickness of the adhesive layer. After the parameter has been applied, the analysis model is established, and the unit properties are assigned after each part is bonded. The model is divided and then meshed, as shown in Figure 12.

#### 2.7.3. Application and Solution of Boundary Conditions for Ice Melting Model

**Figure 13:**
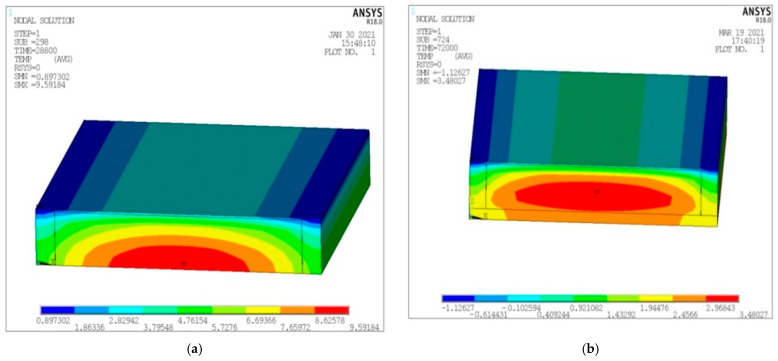
Cloud map of temperature field distribution in melting ice model. (**a**) Temperature field distribution nephogram with insulation layer; (**b**) Temperature field distribution cloud chart without insulation layer.

Firstly, the initial temperature of the model is set to −10 °C. Secondly, the convective heat transfer and voltage are applied. The convective heat transfer is applied to the outer surface of the model in the form of surface load. Depending on the different materials, different convective heat transfer coefficients are set up. The ambient temperature is −10 °C. Zero V is applied on the electrode side and a given voltage is applied on the side. The solution is set to transient analysis. The solution time is set to 8 h (28,800 s). Because the temperature rises slowly without an insulation layer, the solution time is set to 20 h (72,000 s) without an insulation layer. The temperature field distribution nephogram solved is shown in Figure 13.

It can be seen from Figure 13 that the temperature distribution of the melting ice model with and without the heat insulation layer is as follows: the middle temperature at the bottom of the pouring conductive asphalt concrete layer is the highest, and the temperature distribution presents a water wave shape, which increases in turn from inside to out. In the case of the heat insulation layer, the heat is directly transmitted upward, making the temperature of the upper surface increase rapidly. In the case of no heat insulation layer, the heat is transmitted to the steel plate, and some heat is lost. Therefore, the heating rate of the pavement scheme with the insulation layer is faster, and the heating structure with the insulation layer is recommended.

### 2.8. Mechanical Analysis

The composition of the environment and transportation in China is complex, and the overload phenomenon is serious, resulting in a large impact of temperature and load coupling. Therefore, it is necessary to study the internal force of the cast conductive asphalt concrete steel bridge deck pavement system under the coupling effect of snow melting temperature and vehicle load [29].

#### Basic Parameters and Models for Finite Element Analysis of Steel Bridge Deck Pavement

**Figure 14:**
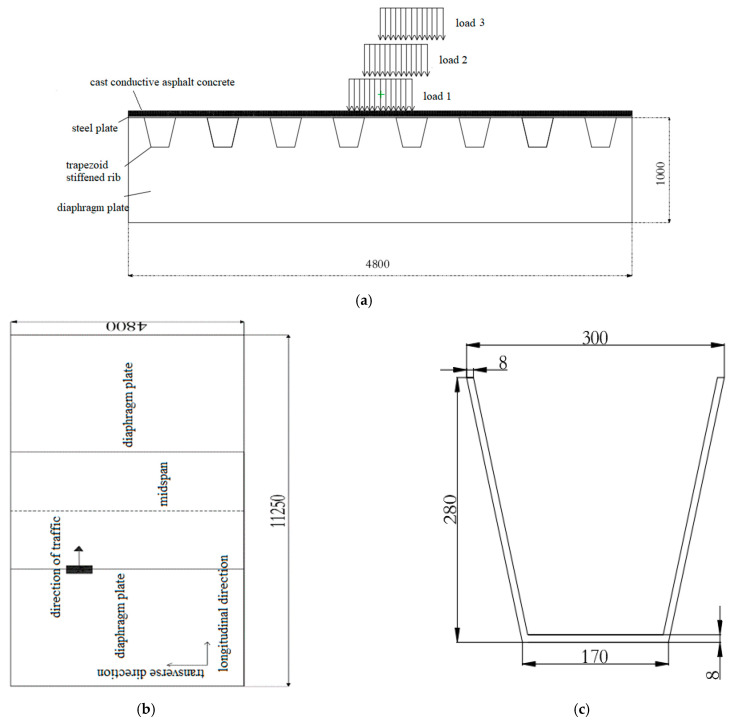
Orthotropic steel bridge deck pavement model diagram (unit: mm). (**a**) cross sectional drawing; (**b**) planar graph; (**c**) trapezoid stiffened rib.

**Figure 15:**
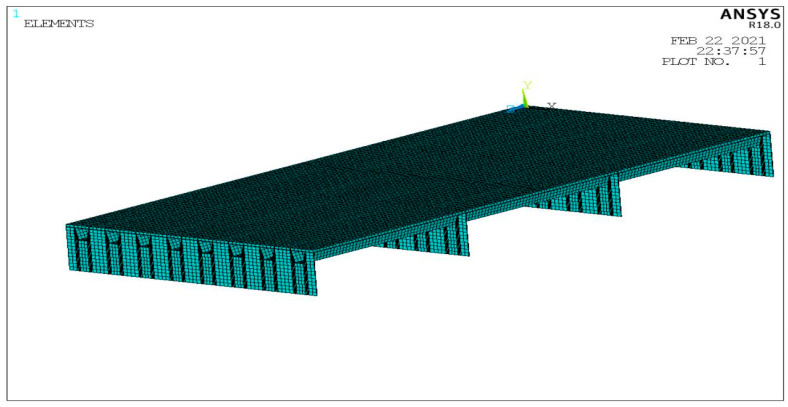
Finite element analysis model diagram.

The mechanical analysis of the orthotropic steel bridges deck pavement is carried out using ANSYS software. The selected orthotropic steel bridge deck plate, trapezoidal stiffener and diaphragm are 16Mnq (low alloy) steel. The density of 16Mnq steel is 7800 kg/m^3^, the elastic modulus is 2.1 × 105 MPa, the Poisson’s ratio is 0.3, the linear expansion coefficient is 1.13 × 10^−5^, the density of CGA-10 is 2500 kg/m^3^, the elastic modulus is 300 MPa, the Poisson’s ratio is 0.3, and the linear expansion coefficient is 2.56 × 10^−5^. The composition of the orthotropic steel bridge deck model selected for analysis is: transverse plate width 4.8 m, transverse bridge with eight trapezoidal stiffeners, longitudinal four diaphragms, plate length 3.75 × 3 m = 11.25 m (see Figure 14 and Figure 15). The size of each component is shown in Table 11.

It has been found that the most unfavorable load position in the longitudinal direction of the orthotropic steel bridge deck is mid-span. In order to obtain the most unfavorable load position in the transverse direction of the bridge under the combined action of temperature and vehicle, the load is transversely added to three special positions in the middle of the mid-span two diaphragms, and the three load positions are distributed as follows:

Load position 1 applies vehicle load as a uniform load to the center of two adjacent stiffeners, load position 2 applies the load as a uniformly distributed load to a uniformly symmetrical positive above the edge of a stiffener as the center, load position 3 applies the load as a uniform load to the center of a stiffener, and the specific load position is shown in Figure 14a. The grounding pressure of the tire is 0.758 MPa. The boundary conditions are fixed at the bottom of the diaphragm, and vertical displacement is allowed without horizontal displacement around the steel plate and pavement. The environmental temperature is −20 °C, and the pavement temperature reaches 4 °C. SOLID70 unit is selected for temperature field analysis, and SOLID185 unit is selected for mechanical analysis.

## 3. Results and Discussion

### 3.1. Influence of Electrode Thickness on Temperature Rise of Specimen

**Figure 16:**
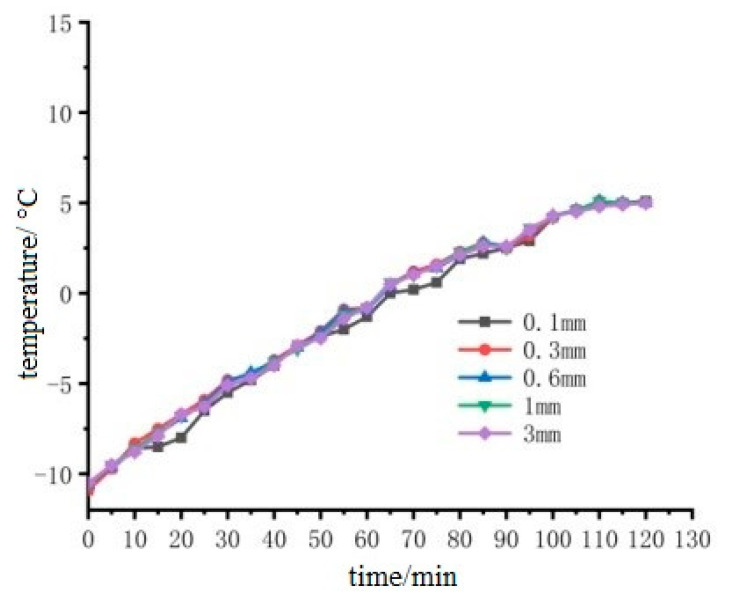
Heating diagram of electrodes of different thicknesses heated for two hours.

**Figure 17:**
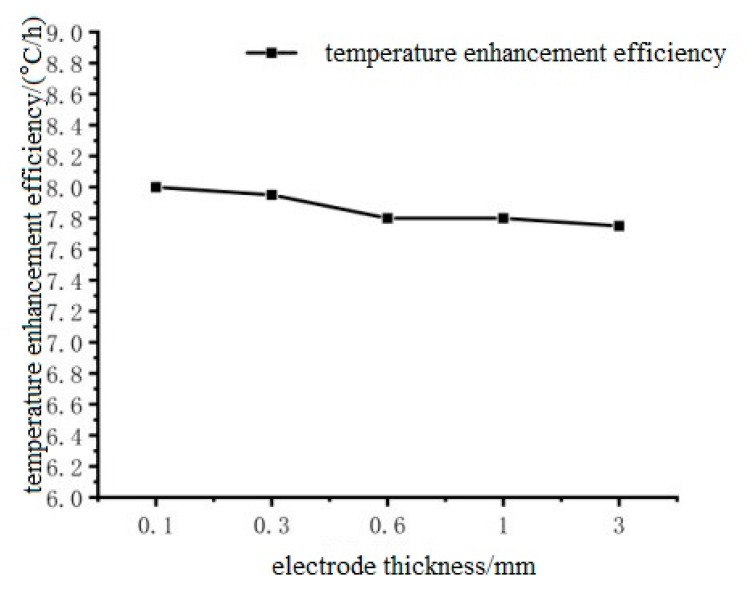
The relationship between electrode thickness and heating rate.

Select the thickness of 0.1 mm, 0.3 mm, 0.6 mm, 1 mm, 3 mm stainless steel electrode patch on the left and right sides of the pouring conductive asphalt concrete heating, and analyze the reasons, the specific heating situation as shown in Figure 16 and Figure 17.

It can be seen from Figure 16 that the temperature-time curve of the middle point of the upper surface of the cast-type conductive asphalt concrete with different thicknesses of external electrodes has the same change trend, and the curve almost coincides. The small fluctuation is due to the start of the refrigeration function, when the temperature of the low temperature box is higher than the set value. The airflow and temperature of the low temperature box show a small change, but this small change does not affect the heating effect of the cast-type conductive asphalt concrete. It can be seen from Figure 17 that in the range of 0.1 mm to 3 mm, with the increase of electrode thickness, the heating rate has an extremely small downward floating. This is due to the increase of electrode thickness, as the rigidity of the electrode and the contact resistance become larger. At the same time, the increase of electrode thickness makes the current flow path longer, which can be explained by ampere law. It can be considered that the electrode thickness has little effect on the heating rate of the cast conductive asphalt concrete when the electrode thickness changes slightly within a certain range, and the influence of the electrode thickness can be ignored.

### 3.2. The Ice-Snow Melting Effect with Insulation Layer

**Figure 18:**
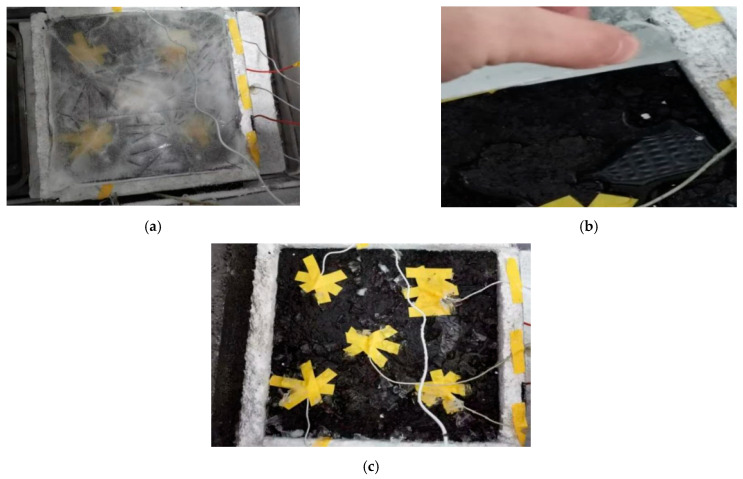
Ice melting test diagram with insulation layer. (**a**) On-test; (**b**) Melting separation; (**c**) Ice melting end.

The ice melting test was carried out on the gating conductive asphalt concrete slab connection power supplies with left- and right-embedded stainless steel metal mesh. The bottom and surrounding of asphalt concrete were wrapped with insulation materials, and the voids were sealed with sealing mortar. The temperature sensors were arranged according to the embedded electrode mode to detect the temperature rise and the starting time of ice melting. When the surface temperature of the gating conductive asphalt concrete was 0 °C, it marked the beginning of ice melting, and the end time of ice melting was observed by visual measurement. A 2 mm-thick ice layer formed on the surface of the asphalt concrete board, which was supported by a tray and placed in a low temperature test box. The temperature of the low temperature box was adjusted to −10 °C, and placed in the water, and the board at the same time froze into ice (12 h). A small opening was opened in the insulation film border to make the melted water flow into the tray. The electrode received a 50 V voltage. The test process is shown in Figure 18.

**Figure 19:**
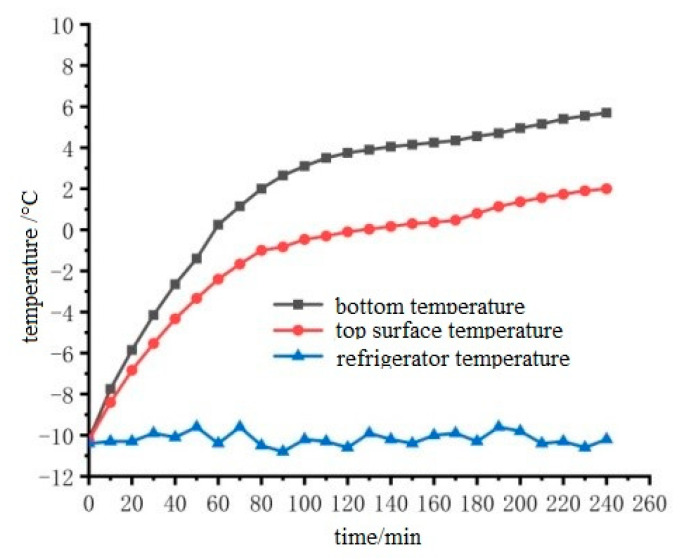
Temperature rise diagram of cast conductive asphalt concrete slab with thermal insulation layer.

In order to verify the snow melting effect of cast conductive asphalt concrete and explore the efficiency of snow melting, it is necessary to consider the temperature distribution of the whole upper surface. To this end, the upper surface temperature was determined by the average temperature of each point on the upper surface. The temperature difference of each point on the upper surface measured by the experiment was between 1 °C and 2 °C. The temperature rise of the upper surface of the cast conductive asphalt concrete slab during the ice melting process was measured as shown in Figure 19.

It can be seen from Figure 19 that as the surface temperature of the specimen gradually rises, the ice and snow begin to melt. From then on, the temperature rise slows down, and the ice continues to melt. When the test was carried out on the middle and posterior sections, the ice layer and the cast conductive asphalt concrete were separated. As shown in Figure 18b, at the end of the test, it was found that, except for a small amount of ice at the edge, the rest of the ice changed from the initial spread of ice and snow to water ice. At that time, it was considered that the ice melting test was over. In the process of ice melting, the lowest temperature of the cast asphalt concrete was about 4 °C. This kind of ice–snow melting method of embedding conductive materials inside the pavement structure has been used in some typical projects, and has achieved good ice–snow melting effect.

### 3.3. Effect of Snow Melting without Insulation Layer

**Figure 20:**
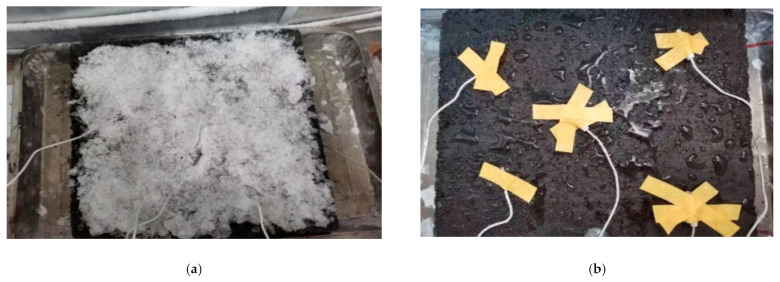
Ice melting test without insulation. (**a**) Ice flower spread; (**b**) Ice flake melting.

The ice melting test of castable conductive asphalt concrete without an insulation layer placed the castable conductive asphalt concrete slab with temperature sensors directly on the tray and in a low-temperature test box. The ice block was formed by 165.6 g of water, broken into ice flakes and evenly distributed on the upper surface of the test block. The temperature of the low-temperature box was set to −10 °C. After 12 h of storage, the slightly melted water was consolidated with the test block again, and the temperature of the whole system reached −10 °C. The test process is shown in Figure 20.

**Figure 21:**
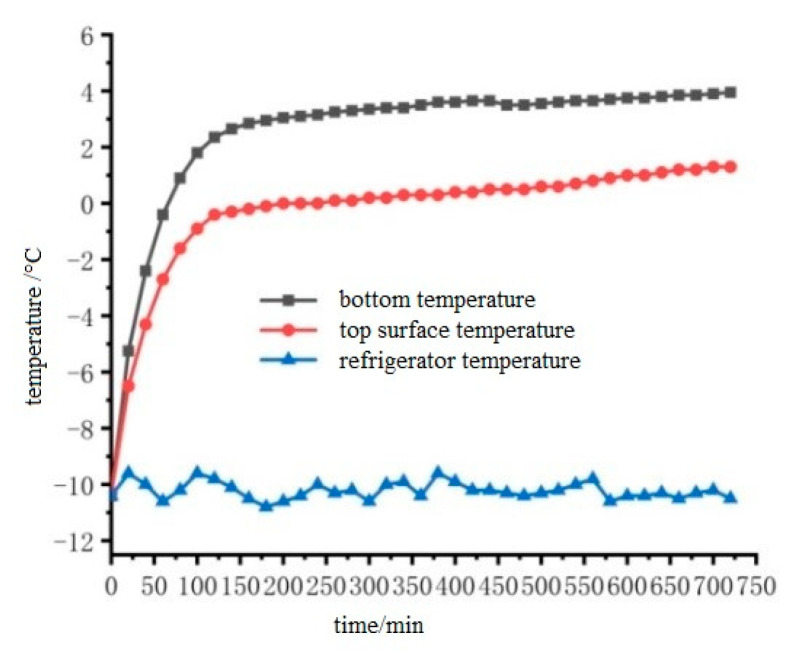
Temperature rise diagram of pouring conductive asphalt concrete slab without barrier.

The average surface temperature and lowest temperature of the cast conductive asphalt concrete during the test was recorded as shown in Figure 21.

It can be seen from Figure 21 that the surface temperature of the small slab of cast conductive asphalt concrete without heat insulation layer rose slowly, and the temperature of the small slab gradually reached 0 °C with the passage of time. At this time, the ice flowers began to melt. During the test, it was found that the melting of the ice flowers on the upper surface was not uniform. The ice flowers in the middle melted first to form a small pool, and the area of melting was the largest. The ice flowers on the edge began to melt after being affected by running water and temperature. The final melting state of the ice flowers is shown in Figure 20b.

### 3.4. Relationship between Ice Thickness, Melting Time and Voltage

**Figure 22:**
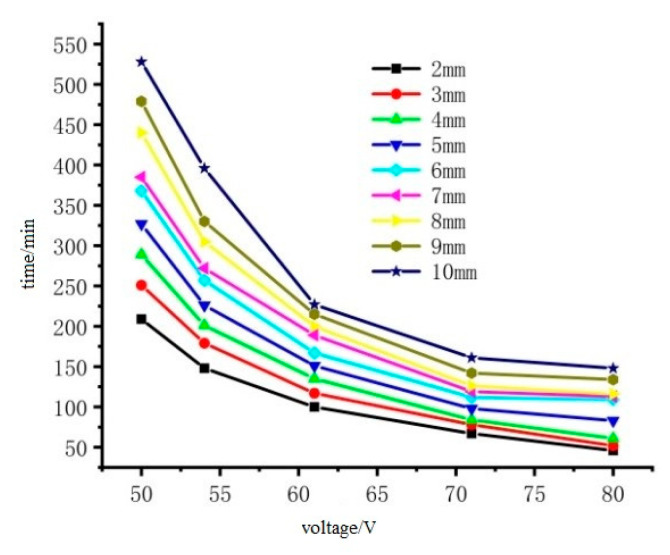
The relationship between ice melting time and thermal insulation and input voltage.

**Figure 23:**
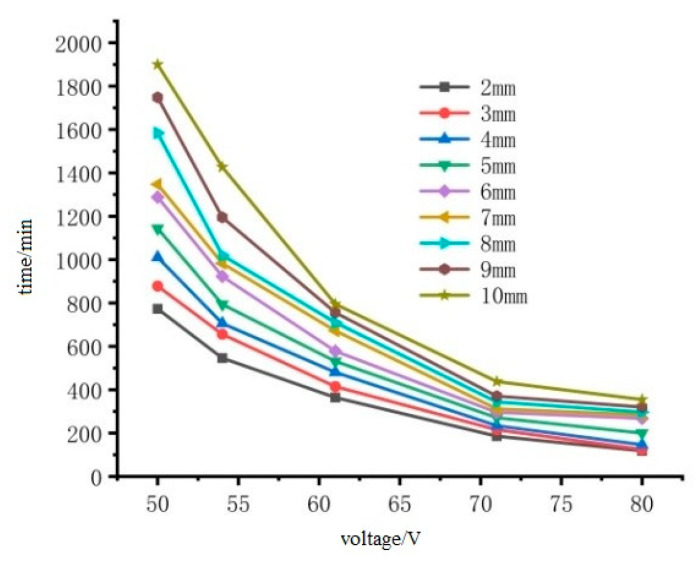
The relationship between ice melting time without heat insulation and input voltage.

The initial temperature of the ice layer was −10 °C. When the surface temperature of the ice layer reached 0 °C, it was considered that the ice melting had ended. Therefore, the temperature of the nodes on the surface of the ice layer picked up, and the time when the temperature reached 0°C was the melting-ice end time. The recommended reasonable input power within the reasonable de-icing time is 400–1000 W/m^2^. In order to more intuitively represent the safety of voltage input, the converted voltage is 50V to 80 V, and the de-icing time under different voltages and different ice thicknesses is obtained, as shown in Figure 22 and Figure 23.

It can be seen from Figure 22 that the melting time of the cast conductive asphalt concrete with the insulation layer became shorter with the increase of input voltage. Considering that the bottom of the pavement layer is steel plate, too much added voltage will affect the durability of the steel bridge and the safety of pedestrians and drivers; the voltage should be below 54 V. If there is a heavy snowstorm, the traffic must be interrupted at this time, and the voltage can be appropriately increased. It can be seen from Figure 23 that the ice melting time without an insulation layer is long. The ice layer with a thickness of 2 mm needed 780 min to melt under the input voltage of 50 V, which was approximately 3.73 times that with an insulation layer. The time difference between the ice melting with and without the insulation layer was large, and the difference became smaller when the input voltage increased. The ice melting time without an insulation layer with thickness of 2 mm under the voltage of 80 V was 119 min, which was approximately 2.5 times that with the insulation layer (46 min). Therefore, in order to melt the ice and snow of the pavement bridge deck more effectively, it is necessary to put a heat insulation layer at the bottom of the conductive layer to isolate the downward transfer of heat. Adjusting the voltage to speed up the speed of ice and snow melting is a better initiative than other methods such as reducing the freezing point of snow. In the process of snowfall change, the ice and snow on the road can be freely melted through energy transformation.

### 3.5. Comparison of Ice Melting Effect with and without Insulation Layer

Compared with the indoor test, the melting time of the finite element simulation was shorter than that of the actual, but the difference was not large. The reason is that the actual ice layer was not evenly heated, the melted water was not discharged in time, and the evaporated water took away part of the heat. The uneven heating of the ice layer made the ice-layer floor void, the heat transfer was slow, and the melting time was prolonged. The combination of indoor test and finite element simulation further verified the effectiveness of cast conductive asphalt concrete ice melting and the reliability of finite element simulation.

By comparing the melting ice of cast conductive asphalt concrete with or without a heat insulation layer, it can be concluded that the melting time of small plate without a heat insulation layer is much slower than that with a heat insulation layer. In the case of no heat insulation, the ice in the middle of the upper surface melts first, and the water flow diffuses outward, which makes the surrounding ice melt slowly, but the duration is relatively long, which is quite different from that with heat insulation. When melting the same thickness of ice and snow, it only takes 240 min with a heat insulation layer, while it takes 720 min without a heat insulation layer, which is 3 times that with heat insulation. Because the ice chips were used in the test without a heat insulation layer, the time required for melting ice and snow without the heat insulation layer may be longer. Therefore, the pavement structure with the thermal insulation layer is more conducive to snow removal and ice removal.

### 3.6. Surface Stress and Strain Analysis of Steel Bridge Deck Pavement

Since the bottom of the pavement layer is steel plate, once the pavement layer cracks, it will affect the performance of the bridge deck. Rainwater infiltration in the thin part of the adhesive layer will rust the steel bridge deck, affecting its service life and strength. Therefore, it is necessary to analyze the tensile stress and strain of the pavement surface to take effective preventive measures.

#### 3.6.1. Longitudinal and Transverse Stress Analysis of Pavement Surface under Different Load Positions

**Figure 24:**
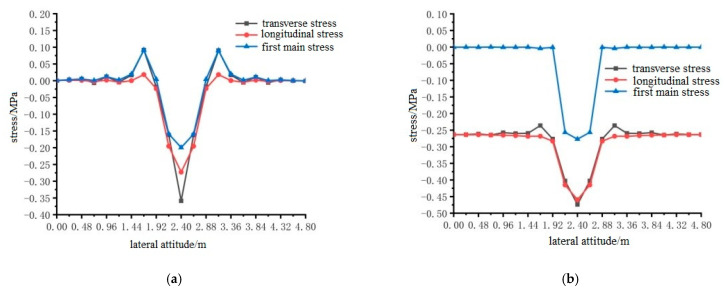
Horizontal distribution of longitudinal and transverse stress on the surface of the pavement with and without temperature field under the action of load level 1. (**a**) Longitudinal and transverse stress diagrams without temperature field; (**b**) Longitudinal and transverse stress diagrams with temperature field effect.

**Figure 25:**
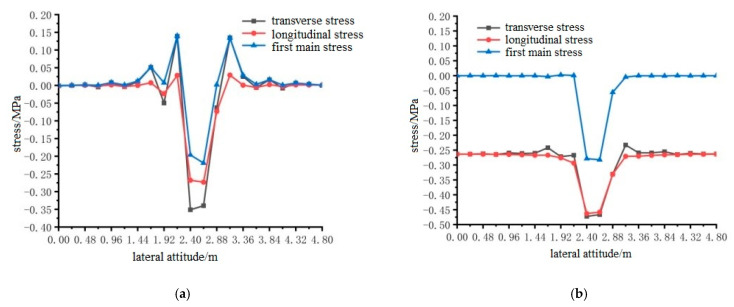
The transverse distribution of longitudinal and transverse stress on the surface of the pavement layer with and without temperature field under the action of load level 2. (**a**) Longitudinal and transverse stress diagrams without temperature field; (**b**) Longitudinal and transverse stress diagrams with temperature field effect.

**Figure 26:**
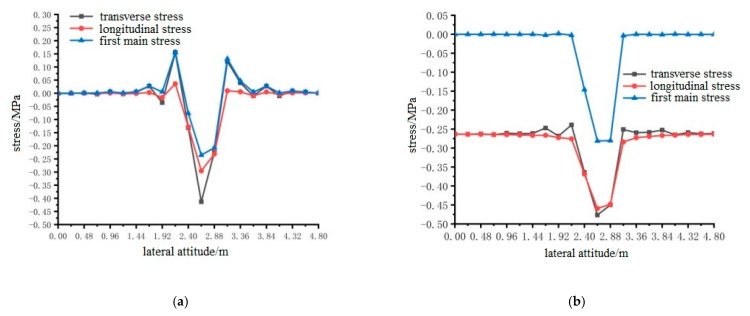
The transverse distribution of longitudinal and transverse stress on the surface of the pavement layer with and without temperature field under the action of load level 3. (**a**) Longitudinal and transverse stress diagrams without temperature field. (**b**) Longitudinal and transverse stress diagrams with temperature field effect.

ANSYS finite element analysis software was used to calculate the longitudinal and transverse stresses on the surface of the pavement layer of the steel bridge under the action of temperature and single wheel uniform load. The calculation results are shown in Figure 24, Figure 25 and Figure 26.

The results shown in Figure 24, Figure 25 and Figure 26 can draw the following conclusions:

1. Under the condition of constant elastic modulus, the transverse maximum tensile stress and compressive stress on the surface of steel bridge deck pavement without a temperature field were much larger than the longitudinal maximum tensile stress and compressive stress. Because the pavement was a three-dimensional space body, the maximum principal stress was not easy to determine, so the transverse tensile stress can be used as an index of pavement design.
2. The maximum tensile stress of the pavement surface under different loading positions were for loading position 1, loading position 2, and loading position 3, 0.3588 MPa, 0.3599 MPa, and 0.4130 MPa, respectively. When the temperature field was applied, the difference of longitudinal and transverse stress values on the surface of the pavement layer decreased, but the transverse tensile and compressive stress values were still greater than the longitudinal tensile and compressive stress values. The maximum tensile stress on the surface of the pavement layer was 0.4511 MPa, 0.4554 MPa and 0.4783 MPa at loading position 1, 2 and 3, respectively, which increased by 25.7%, 26.5% and 15.8%, respectively. Therefore, it can be seen that the temperature field increases the surface tensile stress of the pavement layer. The transverse tensile stress under the action of load position 3 was still the largest, and load position 3 can be used as the most unfavorable transverse load position.
3. The location of the maximum tensile stress in the transverse direction of the pavement surface was 2.4 m, 2.4 m and 2.64 m in the transverse direction of the bridge deck according to the position sequence of the load position. The temperature field had no effect on the location of the maximum tensile stress, but the effect of the temperature field made the maximum compressive stress of the pavement surface smaller.

#### 3.6.2. Longitudinal and Transverse Strain Analysis of Pavement Surface under Different Loading Positions

**Figure 27:**
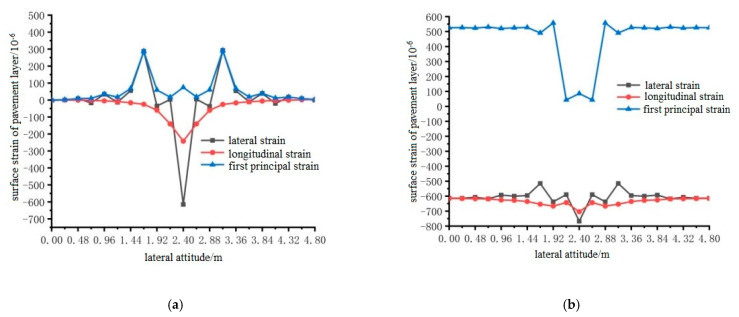
The transverse distribution of longitudinal and transverse strain on the surface of the pavement with and without temperature field under the action of load level 1. (**a**) Longitudinal and transverse strain diagrams without temperature field effect; (**b**) Longitudinal and transverse strain diagrams with temperature field effect.

**Figure 28:**
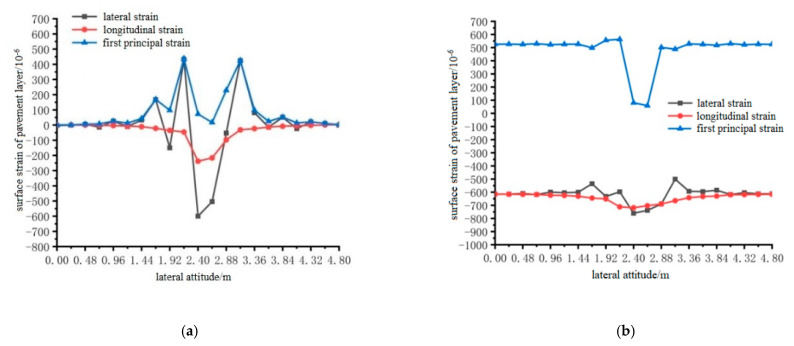
The transverse distribution of longitudinal and transverse strain on the surface of the pavement layer with and without temperature field under the action of load level 2. (**a**) Longitudinal and transverse strain diagrams without temperature field effect. (**b**) Longitudinal and transverse strain diagrams with temperature field effect.

**Figure 29:**
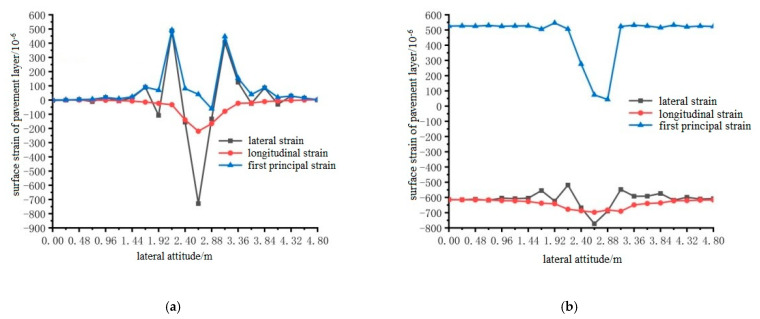
The transverse distribution of longitudinal and transverse strain on the surface of the pavement with and without temperature field under the action of load level 3. (**a**) Longitudinal and transverse strain diagrams without temperature field effect; (**b**) Longitudinal and transverse strain diagrams with temperature field effect.

Using ANSYS finite element analysis software, the longitudinal and transverse strains on the surface of the pavement layer in the middle span of the steel bridge under the action of temperature and single wheel uniform load were calculated. The calculation results are shown in Figure 27, Figure 28 and Figure 29.

From the results shown in Figure 27, Figure 28 and Figure 29, it can be seen that the transverse tensile and compressive strains of pavement surface without the temperature field were far greater than those of longitudinal tensile and compressive strains, and the maximum strain positions appeared in the range of 1.92 m to 3.36 m. Therefore, the maximum transverse strain can be used as a control index of bridge deck pavement design. When the temperature field was applied, the longitudinal tensile strain on the pavement surface increased, and the transverse tensile strain changed little. The compressive strain was mainly reflected in the first main strain, and the first main strain increased. However, the maximum transverse tensile strain was greater than the maximum longitudinal tensile strain. The transverse tensile strain was still used as the design index, and the maximum tensile strain occurred at 2.4 m, 2.4 m and 2.64 m in the transverse direction.

### 3.7. Analysis of Longitudinal and Transverse Shear Stress between Pavement Bottom and Steel Plate

Sliding between the anti-rust adhesive layer and the bottom layer of the asphalt mixture will make the pavement layer and the bridge deck slide. Therefore, it is necessary to select an adhesive layer with good adhesion, to prevent shear failure between layers. Therefore, the shear stress at the bottom of the pavement layer is an important indicator in controlling shear failure.

#### 3.7.1. Analysis of Longitudinal and Transverse Shear Stress between Pavement and Steel Plate under Different Loading Positions

**Figure 30:**
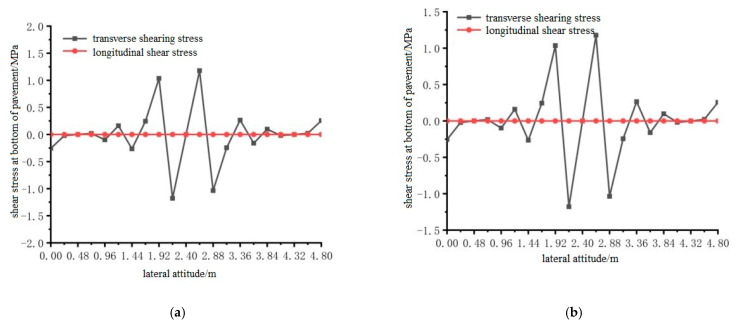
The transverse distribution of longitudinal and transverse shear stress on the bottom surface of the pavement with and without temperature field under the action of load level 1. (**a**) Longitudinal and transverse shear stress diagrams without temperature field; (**b**) Longitudinal and transverse shear stress diagrams with temperature field effect.

**Figure 31:**
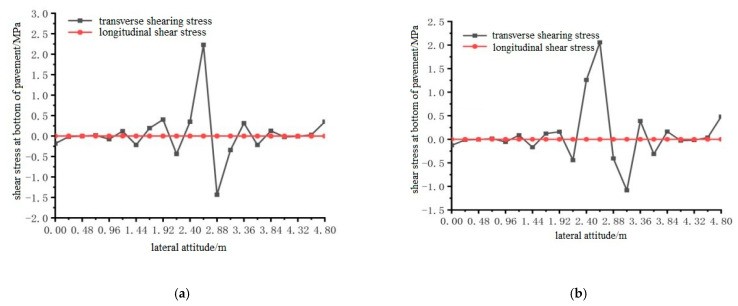
The transverse distribution of longitudinal and transverse shear stress of the bottom surface of the pavement with and without temperature field under the action of load level 2. (**a**) Longitudinal and transverse shear stress diagrams without temperature field; (**b**) Longitudinal and transverse shear stress diagrams with temperature field effect.

**Figure 32:**
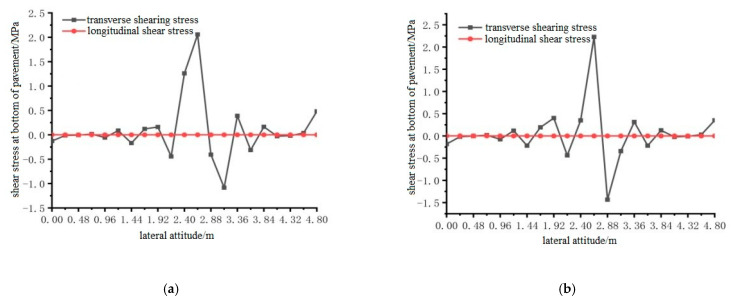
The transverse distribution of longitudinal and transverse shear stress on the bottom surface of the pavement with and without temperature field under the action of load level 3. (**a**) Longitudinal and transverse shear stress diagrams without temperature field; (**b**) Longitudinal and transverse shear stress diagrams with temperature field effect.

Using ANSYS finite element analysis software, the longitudinal and transverse shear stress between the bottom of the steel bridge mid-span pavement layer and the steel plate, under the action of temperature and single wheel uniform load was calculated. The calculation results are shown in Figure 30, Figure 31 and Figure 32.

From the results shown in Figure 30, Figure 31 and Figure 32, it can be concluded that the transverse shear stress at the bottom of the pavement under the action of the temperature field and without the temperature field was much larger than the longitudinal shear stress, and the longitudinal shear stress was very small. The temperature field had no effect on the location of the maximum shear stress at the bottom of the steel bridge deck pavement. The locations of the maximum shear stress under the two conditions are respectively distributed according to the load position: 1.92 m to 2.88 m in the transverse position under the load position 1; the lateral position under load position 2 is 2.64 m; the transverse position of load position 3 is 2.64 m, and the maximum shear stress of load position 2 is the largest among the three load positions.

#### 3.7.2. Longitudinal and Transverse Shear Strain Analysis between Pavement and Steel Plate under Different Loading Positions

**Figure 33:**
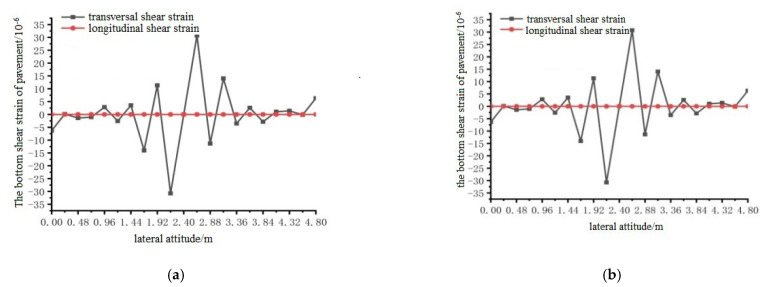
The transverse distribution of longitudinal and transverse shear strain on the surface of the pavement with and without temperature field under the action of load level 1. (**a**) Longitudinal and transverse shear strain diagrams without temperature field effect; (**b**) Longitudinal and transverse shear strain diagrams with temperature field effect.

**Figure 34:**
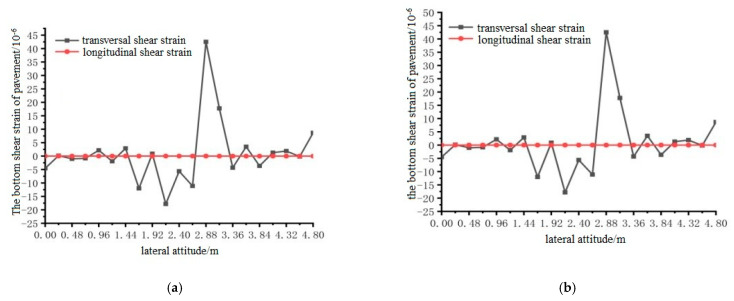
The transverse distribution of longitudinal and transverse shear strain on the surface of the pavement with and without temperature field under the action of load level 2. (**a**) Longitudinal and transverse shear strain diagrams without temperature field effect; (**b**) Longitudinal and transverse shear strain diagrams with temperature field effect.

**Figure 35:**
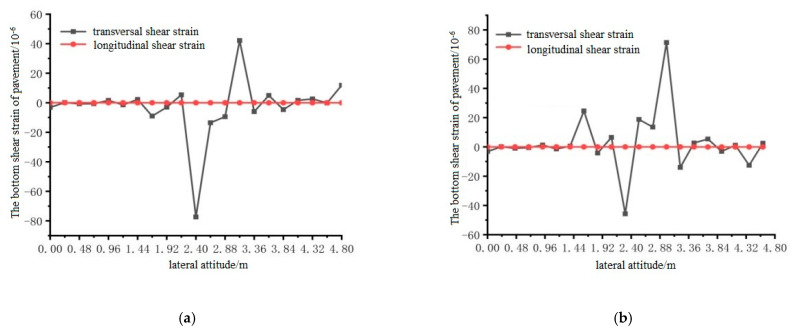
The transverse distribution of longitudinal and transverse shear strain on the surface of the pavement with and without temperature field under the action of load level 3. (**a**) Longitudinal and transverse shear strain diagrams without temperature field effect; (**b**) Longitudinal and transverse shear strain diagrams with temperature field effect.

Using ANSYS finite element analysis software, the longitudinal and transverse shear strains between the bottom surface of the pavement layer and the steel plate in the middle span of the steel bridge under the action of temperature and single wheel uniform load were calculated. The calculation results are shown in Figure 33, Figure 34 and Figure 35.

It can be seen from the results shown in Figure 33, Figure 34 and Figure 35 that the longitudinal shear strain at the bottom of the pavement layer with and without the temperature field was far smaller than the transverse shear strain. The effect of the temperature field made the transverse maximum shear strain increase, but the effect of the temperature field had little effect on the shear strain. The maximum shear strain at position 1 appeared at 1.92 and 2.88 m, the maximum shear strain at position 2 appeared at 2.88 and 3.36 m, and the maximum shear strain at position 3 appeared at 2.4 and 3.36 m.

## 4. Conclusions

1. The stainless steel sheet electrode material used in pouring conductive asphalt concrete has an excellent heating rate, convenient construction, low cost, high safety, and can maintain its stable conductivity during snow melting and ice melting.
2. The electrode layout scheme of pouring asphalt concrete based on melting snow was analyzed. The effects of different electrode layout schemes and electrode thickness on heating rate and snow removal were compared and evaluated. Finally, it was concluded that the buried stainless steel metal mesh was the optimal electrode layout.
3. In the process of finite element simulation of the influence of different voltage inputs on the ice melting thickness, the larger the voltage input, the shorter the ice melting time. With the increase in voltage, the difference in large ice melting time becomes smaller, and the pavement structure with a thermal insulation layer can improve the ice melting efficiency more effectively. The snow melting and deicing time difference between the simulation and the indoor test was short, and the finite element simulation results are credible.
4. The most unfavorable load position of pouring conductive asphalt concrete steel bridge deck pavement under single wheel load and temperature field is mid-span load position 3, and the maximum tensile stress and maximum compressive stress on the pavement surface are transverse. Through comparative analysis, it was found that the maximum transverse stress of the pavement surface increases slightly when the temperature field exists, and the unfavorable loading position remains unchanged. The temperature field has little effect on the low shear stress and shear strain of the pavement.

## Figures and Tables

**Table 1:** Main technical indexes of SBS modified asphalt.

Index		Test Results	Technical Requirements
Penetration (25 °C, 100 g, 5 s)		34	10~40
Softening point/°C		83.5	≮72
Ductility (5 cm/min, 10 °C)/cm		32	≮10
Flash point/°C		266	≮240
Density/(g/cm^3^)		1.05	Actual
After TFOT	Ductility/10 °C	14	≮6
	Quality change%	−0.3	≯±0.5
	Penetration ratio/%	78	≮65

**Table 2:** Main technical indexes of 13.2 mm~2.36 mm gravel.

Index	Test Results 13.2–9.5 mm 9.5–4.75 mm 4.75–2.36 mm			Technical Requirements
Crushing value of stone/%	20.6	--	--	≯26
Apparent specific density/(g/cm^3^)	2.754	2.720	2.779	≮2.60
Hygroscopic rate/%	1.2	1.0	1.5	≯2.0
Needle and plate particle content/%	10.7	10.4	--	≯15
Particle content by washing method/%	0.5	0.7	0.7	≯1
Soft stone content/%	1.9	1.9	2.4	≯3

**Table 3:** Main technical indexes of 2.36 mm~1.18 mm gravel.

Index	Test Results 2.36–1.18 mm 1.18–0.6 mm		Technical Requirements
Apparent specific density/(g/cm^3^)	2.791	2.787	≮2.50
Sand equivalent/%	76	75	≮60
Angularity (flow time)/s	32	32	≮30

**Table 4:** Main technical indexes of mineral powder.

Index		Unit	Determining Value	Technical Requirements
Apparent density		g/cm^3^	2.792	≮2.50
Percent of pass	0.6 mm	%	100	100
	0.15 mm		97.6	90–100
	0.075 mm		91.7	80–100
Appearance		--	no caking	no agglomerates
Hydrophilic coefficient		--	0.9	<1
Plasticity index		%	3.6	<4

**Table 5:** The main properties of graphite.

Index	Shape	Density/ (g/cm^3^)	Particle Size/μm	Carbon Content/%	Ash/%	Iron Content/%	Conductivity/(s/m)
Test results	squamose	2.15	150	98.7	0.2	0.3	3.2 × 10^5^

**Table 6:** The main properties of carbon fiber.

Index	Tensile Strength/MPa	Young’s Modulus/GPa	Density/ (g/cm^3^)	Monofilament Diameter/μm	Carbon Content/%	Filament Length/mm	Conductivity/(s/m)
Test results	4000	240	1.82	10–14	98.0	6–10	2.2 × 10^3^

**Table 7:** Performance indicators of metal electrode materials.

Electrode	Resistivity/(Ω·m)	Price/(yuan/m)
Cu	1.75 × 10^−8^	30
Fe	9.78 × 10^−8^	12
Zn	5.9 × 10^−8^	58
Al	2.83 × 10^−8^	22
Stainless steel	7.42 × 10^−7^	20

**Table 8:** CGA-10 matching requirements [28].

Mesh/mm	13.2	9.5	4.75	2.36	1.18	0.6	0.3	0.15	0.075
Pass rate/%	100	80–100	63–80	48–63	38–52	32–46	27–40	24–36	20–30
Median grading	100	90	71.5	55.5	45	39	33.5	30	25

**Table 9:** Enthalpy values of ice at various temperatures.

Temperature/°C	Enthalpy Value/(J/m^3^)
−40	0
−1	7.33 × 10^−7^
0	3.813 × 10^−8^
10	4.198 × 10^−8^

**Table 10:** Material parameter list.

Material	Cast Conductive Asphalt Concrete	Stainless Steel	Ice
Thermal conductivity/(W·(m·K)^−1^)	2.507	80	2.2
Specific heat capacity/(J/(kg·K))	850	460	2050
Density/(kg·m^−3^)	2500	7850	917
Resistivity/(Ω·m)	3.75	7.42 × 10^−7^	--
Latent heat/ (kJ·kg^−1^)	--	--	336

**Table 11:** The geometric dimensions of each component in the steel bridge deck pavement model.

Length of Steel Bridge Model/mm	11,270	Width of Steel Bridge Deck Model/mm	4800
Height of trapezoidal stiffener/mm	280	Opening width of trapezoidal stiffener/mm	300
Trapezoid stiffener spacing/mm	600	Closed width of trapezoidal stiffener/mm	170
Thickness of trapezoidal stiffener/mm	8	Steel plate thickness of bridge deck/mm	14
Diaphragm spacing/mm	3750	Height of diaphragm/mm	1000
Horizontal diaphragm thickness/mm	10	Pavement thickness/mm	50

## Data Availability

All data that support the findings of this study are included within the articles.
